# An augmented reality home-training system based on the mirror training and imagery approach

**DOI:** 10.3758/s13428-013-0412-4

**Published:** 2013-12-13

**Authors:** Jörg Trojan, Martin Diers, Xaver Fuchs, Felix Bach, Robin Bekrater-Bodmann, Jens Foell, Sandra Kamping, Mariela Rance, Heiko Maaß, Herta Flor

**Affiliations:** 1 Department of Cognitive und Clinical Neuroscience, Central Institute of Mental Health, Medical Faculty Mannheim, Heidelberg University, J 5, 68159 Mannheim, Germany; 2 Department of Psychology, University of Koblenz-Landau, Landau, Germany; 3 Institute of Applied Computer Science, Karlsruhe Institute of Technology, Karlsruhe, Germany; 4 Department of Psychology, Florida State University, Tallahassee, FL USA

**Keywords:** Mirror training, Imagery, Phantom limb pain, Complex regional pain syndrome, Stroke, Rehabilitation, Augmented reality, Virtual reality

## Abstract

Mirror training and movement imagery have been demonstrated to be effective in treating several clinical conditions, such as phantom limb pain, stroke-induced hemiparesis, and complex regional pain syndrome. This article presents an augmented reality home-training system based on the mirror and imagery treatment approaches for hand training. A head-mounted display equipped with cameras captures one hand held in front of the body, mirrors this hand, and displays it in real time in a set of four different training tasks: (1) flexing fingers in a predefined sequence, (2) moving the hand into a posture fitting into a silhouette template, (3) driving a “Snake” video game with the index finger, and (4) grasping and moving a virtual ball. The system records task performance and transfers these data to a central server via the Internet, allowing monitoring of training progress. We evaluated the system by having 7 healthy participants train with it over the course of ten sessions of 15-min duration. No technical problems emerged during this time. Performance indicators showed that the system achieves a good balance between relatively easy and more challenging tasks and that participants improved significantly over the training sessions. This suggests that the system is well suited to maintain motivation in patients, especially when it is used for a prolonged period of time.

**Electronic supplementary material:**

The online version of this article (doi:10.3758/s13428-013-0412-4) contains supplementary material, which is available to authorized users.

## Introduction

Several disorders involving the somatosensory or motor system, such as phantom limb pain, complex regional pain syndrome (CRPS), or hemiparesis after stroke, are thought to be maintained by maladaptive changes in the sensorimotor system. In these conditions, changes in the representation of the sensorimotor areas are observed that covary with the amount of pain or motor disability (e.g., phantom pain, Flor et al., 1995; CRPS, Maihöfner, Handwerker, Neundörfer, & Birklein, 2003; stroke, Liepert, Bauder, Miltner, Taub, & Weiller, 2000). Training methods that provide sensory input to the affected brain region seem to effectively reverse these maladaptive changes. *Mirror training* (Ramachandran, Rogers-Ramachandran, & Cobb, 1995) consists in having patients watch movements of a mirror image of their existing/unaffected limb appearing in the position of their amputated/affected limb. Training over the course of several weeks—partly in combination with additional motor imagery training—has been shown to effectively reverse pain and/or motor disability (Chan et al., 2007; McCabe, Haigh, & Blake, 2008; Mercier & Sirigu, 2009; Moseley, 2004; Thieme, Mehrholz, Pohl, Behrens, & Dohle, 2012), along with a reversal of putatively maladaptive brain changes (Giraux & Sirigu, 2003; MacIver, Lloyd, Kelly, Roberts, & Nurmikko, 2008).

Mirrors or mental imagery can be substituted by virtual reality (VR) or augmented reality (AR) systems. In VR, an interactive simulated visual environment is presented via a head-mounted display (HMD). This may also include body parts capable of replicating natural movement patterns in real time. AR denotes the combination of real-world information with VR—for example, by blending virtual objects into a first-person view video image. In respect to mirror training, these approaches can be used to transpose the movements of the existing/unaffected limb to a simulation of the absent or dysfunctional limb (Eynard, Meyer, & Bouakaz, 2005; Murray et al., 2007; O’Neill, de Paor, MacLachlan, & McDarby, 2003). Such AR- or VR-based mirror-training systems hold several potential advantages, ranging from increased flexibility in the features of the presented limb to the possibility of conveniently and accurately tracking training progress.

The usual mirror-training approaches use arbitrary movements, such as closing and opening the fist, upon verbal instruction. One particularly important feature of VR and AR systems is that they allow the interaction with virtual objects. As a consequence, patients can engage in meaningful movement patterns in more or less self-explanatory tasks.

The aim of the present study was to develop an AR mirror home-training system with a high degree of immersion and identification with the displayed hand. In addition, we developed a set of predefined tasks in the form of computer games, which are interesting and challenging enough to motivate daily practice. The system is intended to aid the treatment of phantom limb pain, CRPS, and impaired motor control and hemiparesis after stroke. This article describes the features of the system and presents an evaluation study conducted with healthy participants.

## Description of the home-training system

**Fig. 1:**
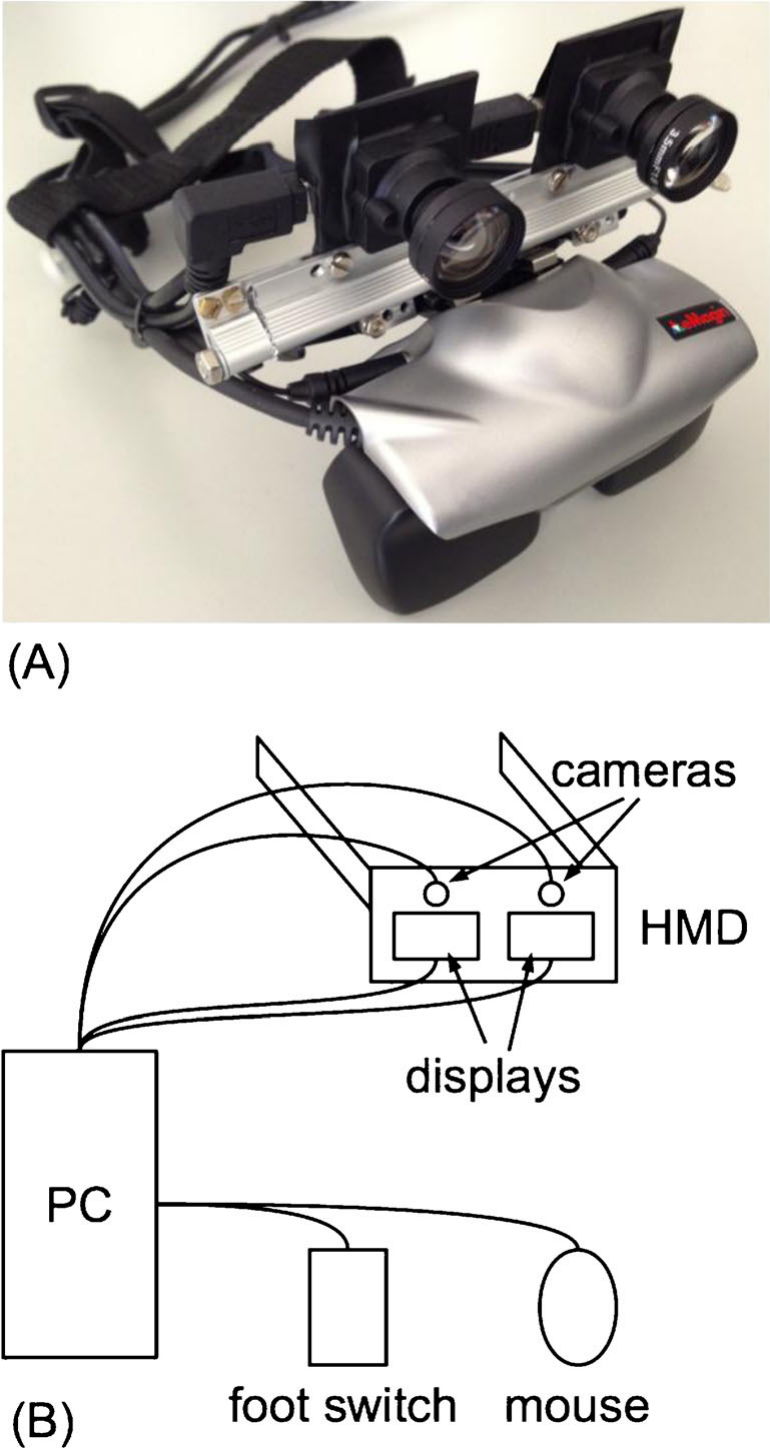
**a** Prototype of the head-mounted display (HMD) based on a commercially available system extended by two cameras. **b** Schematic drawing of all system components. The displays are connected via an interface to the video output; all other components are connected via USB

The home-training system consists of an HMD with integrated cameras connected to a personal computer running custom-made software (Fig. 1).

### Head-mounted display

The HMD is based on a commercially available display (Z800 3DVisor, eMagin, Bellevue, WA) using organic light-emitting diode technology and providing flicker-free images with a resolution of 800 × 600 pixels per eye. The optics of the HMD provide a horizontal field of view (FOV) of 32° and correct geometrical distortions and aberrations. The two displays can be shifted independently in a horizontal direction to adapt to different interocular distances. Two 1.3-megapixel cameras (UI-1642LE-C, Imaging Development Systems, Obersulm, Germany) were mounted on the display using a custom-made fixture made of aluminum. The camera positions could be adapted to the interpupillar distance and the declination angle according to the users’ needs. The camera optics (Lensagon BM3516ND, Lensation, Karlsruhe, Germany) were chosen for low geometrical distortion and low chromatic aberration to ease postprocessing. This setup, in principle, allows stereoscopic 3-D presentations, but this feature was not used in the present version of the training system, which is based on a simpler 2-D version.

### Controlling computer and software

The software was implemented on a personal computer (PC) running a Windows XP Professional operating system. The image data of the camera system were transferred to the PC via the universal serial bus (USB). Then a processed virtual environment was integrated with the image by the software. The combined output was postprocessed and shown on the HMD. The core of the software consists of computer-vision algorithms, which allow the separation of body parts from a unicolor background and detect several hand features like the positions of cardinal points, such as the fingertips (see Bach et al., 2010). The system is capable of either presenting the mirrored limb alone in front of a neutral background or as an overlay of the image captured by the cameras. In principle, the software also allows for separate manipulation of left and right images in order to provide a 3-D presentation. Due to methodological and technical considerations, we did not yet use this feature in the present setup and only processed and displayed images from the left camera to both eyes. However, 3-D will be implemented in future versions. For user interaction, a computer mouse and a foot pedal were connected via USB. The possibility of connecting the computer to the Internet permits the automatic transfer of the training data to a central server.

### User interface

The computer system is set up such that the training software automatically starts after powering up the computer. This eliminates the possibility of system failures due to operating errors. Before the training task begins, the software presents a screen with questions to be answered by clicking on radio buttons with a mouse. With this feature, external measures for monitoring training success—for example, pain ratings—can be obtained and stored along with the training parameters. Then an FOV calibration is performed, ensuring that the user is seated in a position in which only the unicolor background is captured by the cameras. After these preliminary steps, the training tasks start.

### The training tasks

All tasks are based on the general concept of capturing the image of one hand, cropping it from the background, and presenting it in a horizontally mirrored position, mimicking the contralateral hand.

#### Finger flexion task

The finger flexion task is a more sophisticated version of the hand-closing/opening task used in most mirror-training implementations. Specified fingers have to be moved individually, putting more focus on fine motor skills and yielding a more precise vision–motor coupling.

**Fig. 2:**
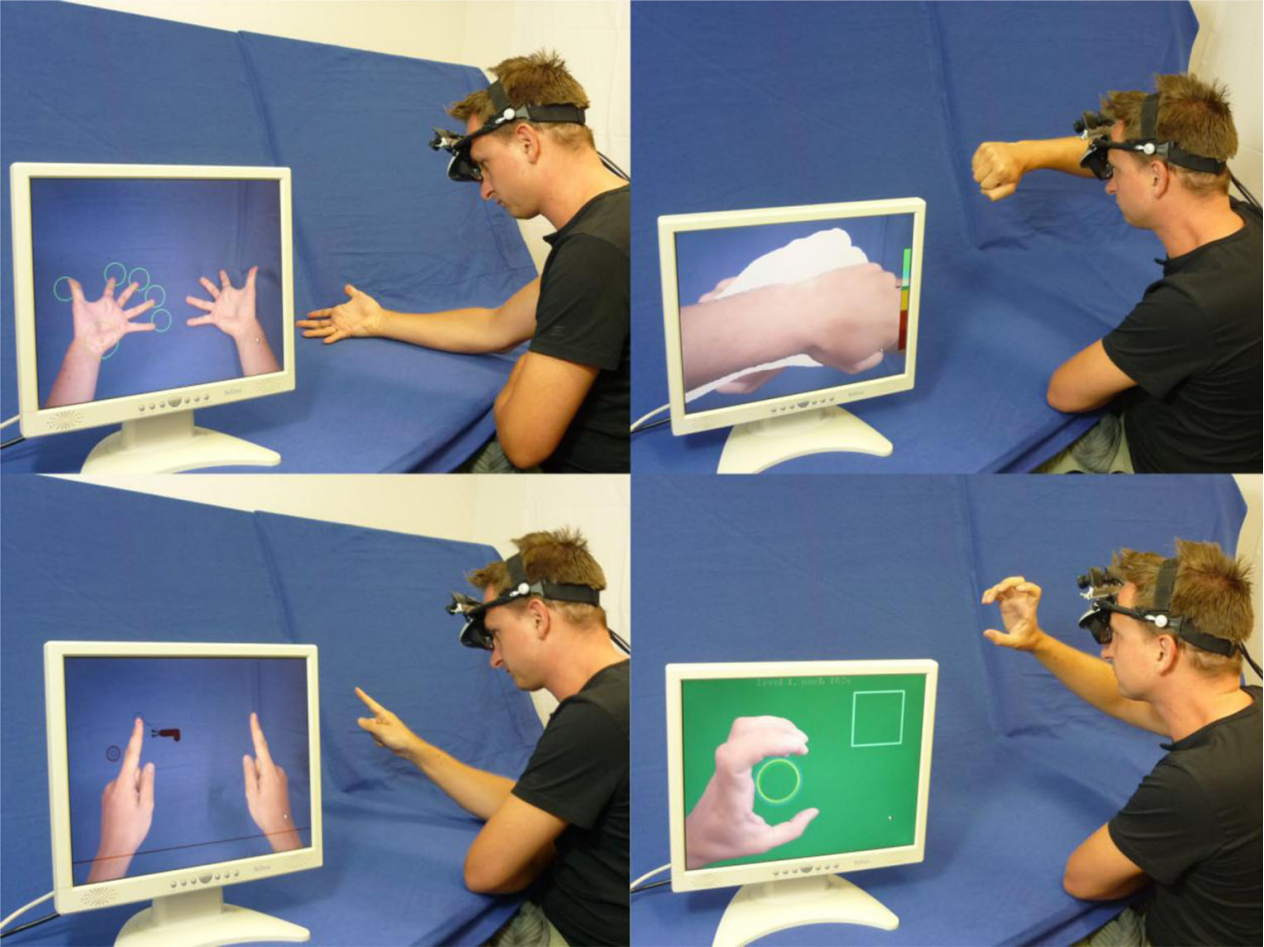
From upper left to lower right: finger flexion task, hand posture task, finger-guided Snake game, ball-grasping task. The images show a participant performing the tasks and an external screen, which is not part of the standard setup, showing the view presented to the head-mounted display. Videos showing the training tasks in more detail are available as Supporting Material on the journal Web site

Participants look in the direction of one hand while seeing a mirrored image mimicking their other hand (Fig. 2a). Participants are instructed to find a comfortable posture for viewing their hand, avoiding unnecessary straining of arm and hand. Once a comfortable posture has been achieved, participants confirm this by pressing a foot pedal. Fingertip positions are then automatically captured by the software, and the task starts.

Open circles surrounding the fingertips are displayed. Each trial of the finger flexion task consists of two phases. In the memorizing phase, participants are required to keep their fingertips within the boundaries indicated by the open circles and watch these circles being filled by green color in a random sequence. The number of fingers, which is indicated in this sequence, is determined by previous performance (see below). Then, in the reproduction phase, participants are to flex individual fingers in the sequence presented in the memorizing phase. As soon as a fingertip leaves its boundary, the circle turns yellow. This allows participants to quickly grasp whether they have indeed flexed the intended finger only. Participants have to keep the finger flexed—and the fingertip outside the boundary—for at least 1 s. Then, the response is accepted, indicated by the boundary circle turning green, and participants can extend their finger so that the fingertip is situated in the boundary again. If the participant manages to flex his or her fingers in the correct sequence, a trial is successfully completed, and a smiley, along with some words of praise, appears. Flexing fingers out of sequence (including accidental flexion of more than one finger at once) immediately cancels the trial and triggers the display of a frowney, along with some uplifting words.

Immediately after each trial, the next trial is started, as long as the predefined duration of 180 s (excluding time for calibration and feedback screens) has not been exceeded. After a successful trial, the length of the finger flexion sequence is increased by one more item; after an unsuccessful trial, its length is reduced by one item. After the last trial, a clock icon appears, along with a message stating that the training time is over.

#### Hand posture task

In the hand posture task, patients have to combine mental imagery with extensive movements of the whole arm and fine movement skills of the fingers.

Target hand postures are presented as white silhouettes, similar to an inverted shadowgraphy game (Wikipedia contributors, 2012b). The participants’ task is to move their mirrored hand into a posture fitting the silhouette. In order to solve the task, a predefined amount of overlap between the arm projection and the silhouette has to be achieved. At the right side of the display, a color-coded scale indicates the amount of overlap. Once the overlap criterion has been reached, the trial is solved, a smiley along with some words of praise appears, and then the next trial begins. In total, the set consists of 41 different silhouettes of varying difficulty, ranging from a closed fist to very complex forms. Each training session starts with an easy trial; then silhouettes are picked at random by the software. Participants are given a maximum time of 30 s to complete one trial. If they do not succeed within this period, the trial is counted as unsuccessful, and the next trial starts. This feature was included to prevent frustration arising from being “stuck” with very difficult silhouettes for the whole training period. New trials are started until the net training duration of 180 s has been exceeded. This means that if a new silhouette appears after a net training duration of 150 s, the user still has 30 s to finish the task. This can extend the training time for a maximum of 29 s, depending on the starting time of the last silhouette.

#### Finger-guided Snake game

This task focuses on moving the hand in space with shoulder and elbow movements while the finger and wrist are kept mostly rigid.

In this classical Snake game (Wikipedia contributors, 2012a), the participants’ task is to guide a virtual snake to virtual cookies. The snake follows the extended index finger of the mirrored hand. The speed of the snake can be controlled via the distance between snake and finger: The greater the distance, the faster the snake moves. Care has to be taken that the snake does not “bite” itself: Its “head” must collide neither with its “tail” nor with the screen’s borders. With each successfully “eaten” cookie, difficulty is increased by elongating the snake. After unsuccessful trials—the snake “biting” itself or moving outside the display boundaries—difficulty is decreased. New trials are started until the net training duration of 180 s has been exceeded.

#### Ball-grasping task

In the ball-grasping task, task, elbow and shoulder movements, as well as fine movement skills of the thumb and index finger, are trained.

The goal is to grasp a ball by forming a “C” with the thumb and index finger and carry it to a quadratic target area, where it has to be slightly squeezed in order to finish the trial. As in the other tasks, participants perform movements with one hand while seeing its mirrored image. Each contact with the ball induces movement, resembling a rubber ball: After being pushed, it rolls away, and even if the ball has been caught, it may “wriggle” out again if the thumb and index finger are held too far apart. If the ball is squeezed too tightly or if it is pushed too hard, it changes color as a warning signal; if the force becomes too high, it eventually pops. New trials are started until the net training duration of 180 s has been exceeded.

## Evaluation of the training system

We evaluated the system in a small sample of healthy participants. In this pilot study, we were mainly interested in ensuring that the tasks are neither too easy nor too difficult in order to achieve a high motivation for using the system over a longer period of time.

### Method

#### Participants

A total of 7 naïve participants (1 male, 22–53 years of age, all right-handed) recruited from the staff and interns of the Department of Cognitive and Clinical Neuroscience, Central Institute of Mental Health, took part in the study.

#### Procedure

Six participants completed a total of 10 training sessions, 1 completed only 9 training sessions; 3 participants trained once a day, and 4 participants trained twice a day, all with their right hand. The mirrored image of the hand was displayed against a neutral background in all tasks.

For the hand posture task and the ball-grasping task, performance was indicated by the number of successfully completed trials per session. In the finger flexion task and the Snake task, the difficulty could vary between trials and was expected to increase over the course of the training. Therefore, we used the number of successfully performed trials per session multiplied by the average difficulty of the task (i.e., fingers to be moved and length of the snake, respectively) as performance indicators.

These performance indicators were analyzed longitudinally with linear regressions, and Wilcoxon signed rank tests were used to compare performance between the first and the last sessions. All computations were performed with R 2.14.1 (R Development Core Team, 2012).

### Results

**Fig. 3:**
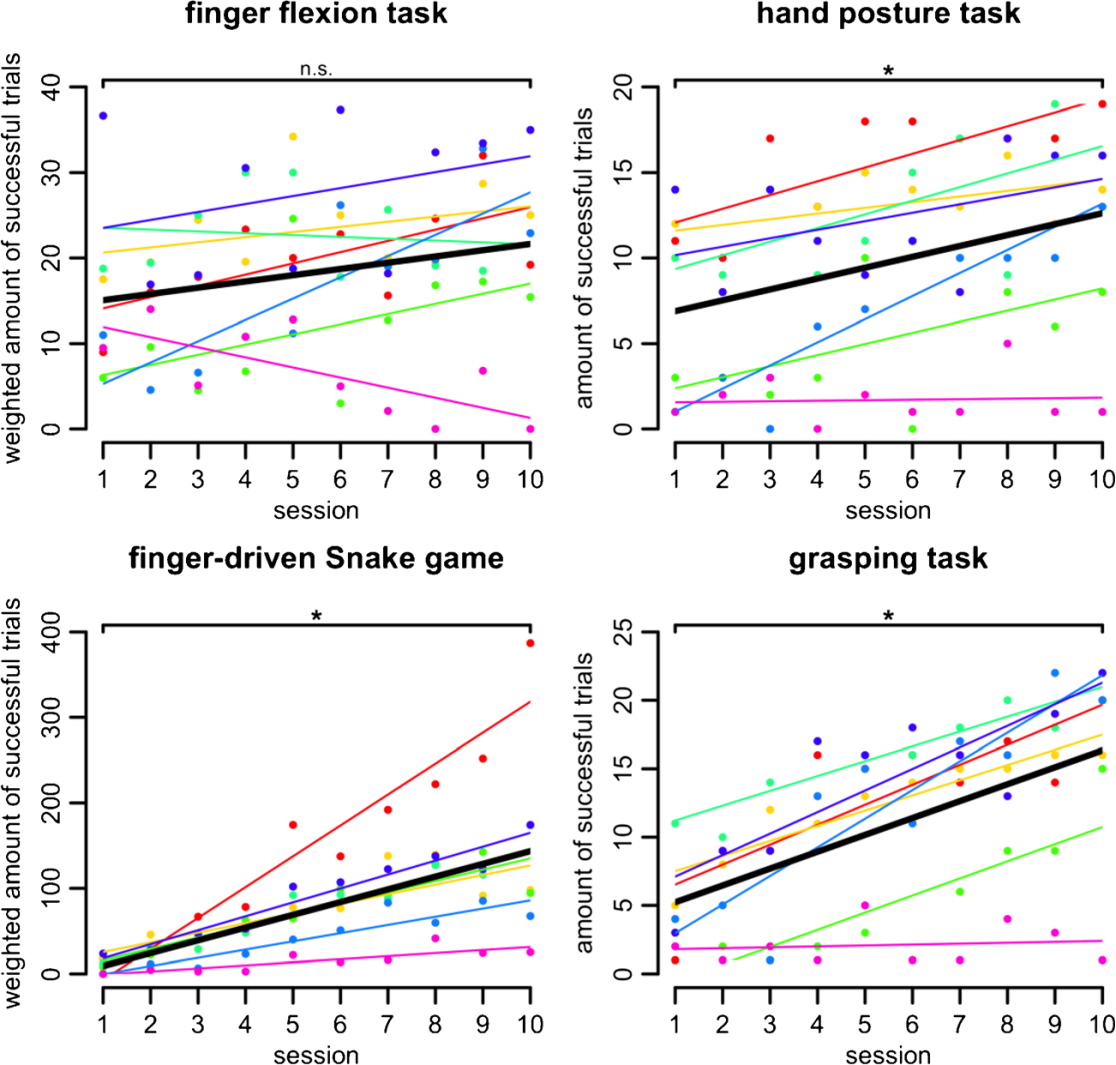
Session numbers are shown on the *x*-axis. The *y*-axis shows the amount of successful trials (hand posture task, grasping task) or the amount of successful trials weighted by difficulty (finger flexion task, finger-driven Snake game; see the Method section for details). Colored dots show the individual score per training session. Colored thin lines show the individual linear regressions over theses scores. Thick black lines show the linear regression on the group level based on the average intercept and slope parameters of the individual linear regressions. Brackets indicate the results of one-tailed Wilcoxon signed rank tests between the first and last sessions (n.s., not significant; *p < .05)

Most participants showed increases in performance over the 10 training sessions, as indicated by the positive slopes of the linear regressions from the performance measures on the session number (Fig. 3). On the group level, this was confirmed by significant differences between the first and the last sessions in three of the four tasks (one-tailed Wilcoxon signed rank tests; hand posture task, *V* = 1.5; Snake game, *V* = 0; ball-grasping task, *V* = 1; all *p*s < .05).

The finger flexion task was an exception because, here, no substantial increase in performance could be detected on the group level over the course of the training sessions (one-tailed Wilcoxon signed rank test, *V* = 8.5, n.s.). This may have two different reasons. First, in this task, performance is restricted both by short-term memory span and by finger flexion speed, thus leaving much less space for improvement than the other tasks. Second, performance in the finger flexion task crucially depended on the hand’s baseline posture and position recorded at the beginning of the task. If this original position was awkward and hard to maintain, the possibility of successfully performing the task was severely impeded. Future versions of the software will allow recalibrations to avoid this situation.

## Discussion

We developed an AR-based home-training system based on the mirror and imagery training approach suitable for the treatment of phantom limb pain, CRPS, and impaired motor control after stroke. The system was evaluated in a sample of healthy participants and shown to function flawlessly. We also assessed and analyzed performance data from the individual tasks and found significant performance increases in most participants and tasks.

One main advantage of VR and AR training systems over the classical mirror or imagery approach is a tight control over what patients actually do during training. Not only can the tasks be implemented in a standardized way, but also these systems are capable of automatically assessing numerous parameters for tracking performance and compliance. One clear benefit of the classical real-life mirror-training approach over VR implementations is the close resemblance of the mirrored hand to one’s own amputated or impaired hand. With the system presented here, we can use the mirrored image of one’s own hand and use it in the context of a simulated environment. This combines the methodological advantages of VR systems with the verisimilitude of real-life mirror or imagery training.

The system presented here has high adaptability. The captured image of one’s own hand can be processed in several ways before presenting it on the HMD. This feature is important for several clinical applications: Many amputees perceive their phantom as being shrunken, as compared with their former existing limb (“telescoping”; Jensen, Krebs, Nielsen, & Rasmussen, 1983), and this phenomenon seems to be related to the extent of PLP (Grüsser et al., 2001). CRPS patients often have swollen limbs or perceive them as being larger than they actually are (Moseley, 2005). The real-life mirror or imagery training does not account for these phenomena and may fail to achieve spatial congruence between the mirrored image of the intact limb and the percept of the amputated or impaired limb. With the AR setup, however, the “mirrored” image can be scaled, altered, and shifted to yield an optimal fit. Furthermore, there are reports that pain can be reduced by a scaled-down visual feedback of the affected limb (Moseley, Parsons, & Spence, 2008). Exploiting this effect may be beneficial for the learning mechanisms assumed to underlie mirror and imagery training. In imagery training, the ability of the participant to immerse her- or himself into the imagined movement is crucial, and here the described AR approach can also help in visualization.

In the setup presented in this article, we did not use the possibility of inducing spatial visual perception via stereoscopic presentation, because we wanted to develop a simple and easy-to-use system. To name just a few challenges, the necessary postprocessing steps would have to be conducted in parallel for both images; presenting stereo images harbors the risk of flickering, and the individual adjustment of cameras and displays becomes more complicated. Therefore, for this first version of the system, we settled on presenting the image captured with the left camera to both eyes. A 3-D version, however, is in preparation.

Perhaps the most obvious potential for improvement lies in transferring the system to less extensive hardware. The currently used setup was chosen to warrant high processing speed and image quality. With the steady increases in processing and graphics performance, we plan an implementation on netbooks or tablet computers for future versions.

## Supplementary Online Material

These videos show the four tasks used in the home-training system. They are available from the start screen of the system, so that users can refer to them if they need to be reminded how exactly the tasks have to be performed,<https://dl.dropboxusercontent.com/u/25017149/finger_movement_task.avi>.

## Electronic supplementary material

Below is the link to the electronic supplementary material.

ESM 1 Finger flexion task 8.78 MB

ESM 2 Hand posture task 7.74 MB

ESM 3 Finger-guided Snake game 3.70 MB

ESM 4 Ball grasping task 5.96 MB
